# Effect of Physical Activity and Exercise on the Level of COVID-19 Antibodies and Lifestyle-Related Factors among Vaccinated Health Science Center (HSC) Students: A Pilot Randomized Trial

**DOI:** 10.3390/vaccines10122171

**Published:** 2022-12-16

**Authors:** Abdulaziz M. F. Shayea, Naser M. Alotaibi, Mohammed Shaban Nadar, Kawthar Alshemali, Hussah W. Alhadlaq

**Affiliations:** 1 Department of Occupational Therapy, Faculty of Allied Health Science, Kuwait University, P.O. Box 24923, Kuwait City 13110, Kuwait; 2 Department of Molecular Biology, Faculty of Graduate Studies, Kuwait University, P.O. Box 24923, Kuwait City 13110, Kuwait; 3 Department of Environmental and Occupational Health, College of Public Health, Kuwait University, P.O. Box 24923, Kuwait City 13110, Kuwait

**Keywords:** vaccine, physical activity, antibodies, lifestyle

## Abstract

A vaccine is a type of medicine that increases immunity and the number of antibodies (IgM and IgG) when injected into the body, preparing it in case of an actual viral infection. It has been shown in several studies that there is a significant relationship between physical activity and vaccination. Furthermore, it has been documented that physical activity can play a major role in reducing stress. Evidence also shows the existence of a relationship between immunity, vaccine response, and sleep duration. To investigate the effects of physical activity on the level of COVID-19 antibodies and lifestyle-related factors, Health Science Center (HSC) students who had taken the third dose of the vaccine and had no prior infection of the COVID-19 virus were investigated. To serve the purpose of this study, an anti-SARS-CoV-2 test was applied by taking a blood sample from the students. The Perceived Stress Scale (PSS) and Pittsburgh Sleep Quality Index (PSQI) questionnaires and the Borg’s 15-point scale were given to the participants to fill out. The study utilized a two-arm randomized control research design in which 40 participants were randomly assigned into one of two groups, either the control group (*n* = 20) or the treatment group (*n* = 20). All tests and assessments were performed before and after intervention for both groups. The control group walked less than 5000 steps every day for one month with a 20 min rest during the exercise session, while the treatment group walked more than 12,000 steps every day for the same time and exercise task session. The students’ steps were monitored using an Apple watch. There was a significant decrease in the IgG antibody level in the treatment group compared to the control group (*p* < 0.001). The IgM antibody level of all groups did not show any significant difference before starting the intervention. However, there was a significant (*p* < 0.05) decrease in the IgM level of the treatment group after treatment compared to before treatment. Moreover, there was a significant decrease in the treatment group’s stress level and sleep disruption, indicating better sleep quality, compared to the control group (*p* < 0.035). The levels of IgG and IgM did not improve for the treatment group. However, the treatment group improved their stress level and sleep disruption. Therefore, further rigorous research is needed to investigate vaccine efficacy among more physically active people.

## 1. Introduction

The current global COVID-19 pandemic has severely impacted health-related factors. There are several groups of people who are considered at high risk of SARS-CoV-2 infection [1]. Exercise improves mitochondrial health, and healthy mitochondria are necessary for proper immune function [2]. However, during the pandemic, many people have become less active, consumed unhealthier food, gained weight, and experienced an overall decline in physical health, which, in turn, may have contributed to reduced immunity [3]. To counter such effects, healthcare professionals have recommended engaging in healthy home programs, home exercise, and a healthy diet [4]. Furthermore, many lifestyle routines have changed since the outbreak of the pandemic and its subsequent lockdowns, especially sleep patterns and sleep quality [5]. The COVID-19 vaccines approved by the WHO include Pfizer-BioNTech, Oxford/AstraZeneca, Johnson and Johnson, Moderna, Bharat Biotech, Sinopharm, and Sinovac. However, Oxford/AstraZeneca, Pfizer-BioNTech, Johnson and Johnson, and Moderna are the vaccines used in Kuwait. Pfizer and Moderna use messenger RNA (mRNA) to deliver a message to the immune system that tells the cells to create a spike protein foreign to the body, causing the immune system to attack it by creating antibodies and making the immune system prepared for any future conduction of the virus [6]. A lower risk of infection and a greater immunity can indeed be received through a regular and active lifestyle for all ages. Multiple studies during the pandemic have investigated whether the body’s physical activity relates to the vaccine’s response. In a recent study, two groups of elderly women who were vaccinated using the influenza vaccine were included in a study [7]. The first group included women who walked 18,000 steps daily for two weeks, while the second group included women who walked less than 10,000 steps daily for two weeks. A greater immune response was observed in the first group compared with the second group after two days of receiving the vaccine. Furthermore, a greater adaptive response was also spotted in the first group after one week of vaccination. In another study that compared older men who were physically active and vaccinated by influenza vaccine, the participants had engaged in regular aerobic exercises three times a week for the past two years compared to older men who had absolutely no exercise for at least three times a week for the past two years, with the results highlighting a higher level of antibody response in the active men compared to the inactive men [8]. Similarly, Kohut et al. reported that adults 62 years old and above who participated in at least 20 min of intense exercise three times or more a week showed a significantly higher antibody response after receiving the influenza vaccine than the same age group who had an inactive lifestyle [9]. Sleep quality is another factor that is known to increase the immune system and facilitate one’s ability to stay active and strong. In one study that investigated the effect of sleep on immunity and vaccine response, a comparison was conducted between two groups of young adults who took the hepatitis A vaccine. One group had a regular night’s sleep, while the other group was not allowed to sleep for 36 h after vaccination. The results showed that the group with regular sleep had a higher immune response compared to the other group [10].

Having said that, physical activity affects the levels of COVID-19 antibodies, stress, and sleep quality. In addition, to the best of our knowledge, there are no previous studies that have investigated the effects of physical activity on the level of COVID-19 antibodies and lifestyle-related factors following the third dose of the COVID-19 vaccine. Therefore, our study objective was to investigate the effect of physical activity on COVID-19 antibodies (IgG and IgM), stress, and sleep quality (lifestyle-related factors) in Health Sciences Center (HSC) students who had taken the third dose of the COVID-19 vaccine and had no prior COVID-19 infection. Several confirmation tests were performed. A rapid antigen test, which can provide false negative results, was applied. However, these false negative results were avoided by using the antibodies test level, patients self-report, and chest x-ray, which was used to investigate any past infiltration in the lung.

## 2. Methodology

### 2.1. Participants

**Figure 1:**
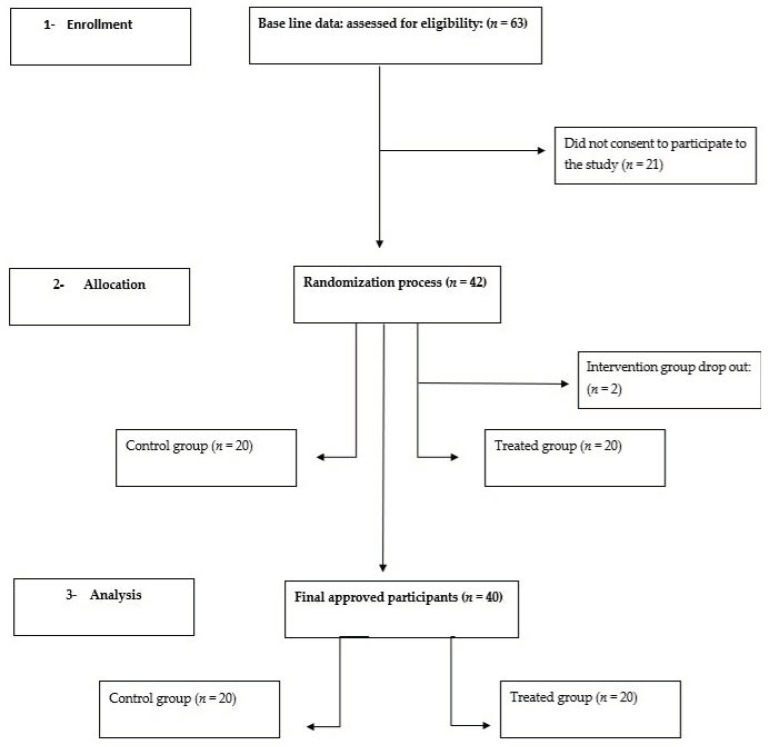
Study flow diagram.

Forty (HSC) students were randomly selected from a student registry list. The inclusion criteria included no prior infection of COVID-19, having taken two doses of the COVID-19 vaccine and intending to take the third dose of the vaccine. Students who had chronic illnesses, such as diabetes or heart disease, were excluded from the study. Randomization was completed by using a computer-generated random number sequence. Then, the students were randomly distributed into either Group 1 (control group; *n* = 20) or Group 2 (treatment group; *n* = 20). The treatment was based on exercise and walking more than 12,000 steps every day for one month, whilst the control group walked less than 5000 steps every day for the same amount of time (see (Figure 1)).

### 2.2. Study Design and Instruments

This randomized controlled trial utilized the following standardized outcome measures: Perceived Stress Scale (PSS-10), Pittsburgh Sleep Quality Index (PSQI), and Borg’s 15-point scale. These assessments were validated outcome measures to demonstrate the effectiveness of the intervention. The Perceived Stress Scale (PSS-10) screens for stress experienced during the past month, whilst the Pittsburgh Sleep Quality Index (PSQI) screens for sleep quality. Meanwhile, Borg’s 15-point scale screens the exercise intensity. All of them are standardized self-reported questionnaires [11,12]. For more elaboration, the purpose and administration of these assessments were explained in administration of assessments and perceived exertion and heart rate sections respectively.

Apple watches were used to monitor the students’ steps per day, as it is an accurate device for counting steps and estimating heart rate [13]. The apple watch was lent to the participants and they had to return it at the end of the study.

### 2.3. Blood Collection

Blood sample was taken from an antecubital vein (30 mL) of the subjects just before taking the third dose of the vaccine, and again at four weeks post-immunization after intervention.

### 2.4. Anti-SARS-CoV-2 Test and Antibody Level

A GenScript (Genscript Biotech Corp, Piscataway, USA) SARS-CoV-2 Spike S1-RBD IgG&IgM ELISA Detection Kit was used to measure the antibody levels. The manufacturer’s directions were followed, where serum was diluted at 1:100 and HRP-conjugated mouse anti-human IgG Fc was added to the plate to detect anti-RBD [14].

### 2.5. Procedure

Before data collection, ethical approval was obtained from the institutional review board (IRB) of Kuwait University. The date of getting ethical approval was 11/4/2022 and the identification number was 567. To measure the baseline level of IgG and IgM antibodies, an anti-SARS-CoV-2 test was applied for both the control and treatment groups by taking blood samples from the students before taking their third dose of the COVID-19 vaccine and before starting the intervention. The Perceived Stress Scale (PSS-10) and Pittsburg Sleep Quality Index (PSQI) questionnaires were given to the participants to fill out before the beginning of the intervention. Additionally, Borg’s 15-point scale was used. After obtaining their baseline antibody level, the students took their third COVID-19 dose and started the intervention immediately. A 15 min exercise task and a goal of 12,000 steps were given to the treatment group, whereas 20 min rest and 5000 steps were allocated to the control group. All of the mentioned tests and assessments were performed once before the intervention before taking the third dose, and a second time after the third dose and after a month of following the intervention for both groups.

### 2.6. Exercise Task and Walking Steps

Elastic resistance bands were used in the exercise task for the treatment group. This exercise was performed in sets of 30 s of exercise. After this, the participant rested for 30 s. Three exercise movements, namely, a lateral raise, an upright row, and a chest press, were performed by the participant. Motivation and encouragement to perform the exercise were provided. The participants alternated movements and performed each five times by completing 15 min of exercise. To maintain exercise for 30 s, the resistance band strength was adjusted to remain challenging while still achievable [15]. Regarding walking steps, Group 1 (control group) was instructed to walk less than 5000 steps a day for one month whilst Group 2 (treatment group) was required to walk over 12,000 steps a day for one month as well. The students’ steps were monitored using an Apple watch and they had to provide a screenshot of their daily step count to the first author electronically [13].

#### Perceived Exertion and Heart Rate

Perceived exertion was recorded during the exercise task. This was achieved by providing the participants the Borg’s 15-point scale immediately after each set as it is shown in (Table 1) [16]. Their heart rate was recorded using an Apple watch [13].

### 2.7. Administration of Assessments

#### 2.7.1. Sleep Quality

The Pittsburg Sleep Quality Index (PSQI) is a standardized tool that can measure the quality and patterns of sleep issues in seven domains of sleep. The participants were instructed to answer all of the questions based on subjective views and rate them using a four-point Likert scale, with 0 indicating good sleep quality, 1 fairly good sleep quality, 2 fairly bad sleep quality, and 3 bad sleep quality. The seven domains of concern were covered by the questions randomly, meaning that some of the concerned areas had more than one question. The researchers followed the standardized scoring criteria by summing the responses corresponding to each domain separately [17].

#### 2.7.2. Stress

The Perceived Stress Scale (PSS-10) is a self–reported questionnaire for measuring psychological stress and how individuals perceive their life situations as stressful [18]. The questionnaire evaluates how much the respondents believe their life has been unpredictable, uncontrollable, and overloaded in the last month. The PSS-10 has good internal, convergent, and concurrent validity, and is sensitive to measuring perceived stress [19]. The scale consists of six negatively stated items (items 1, 2, 3, 6, 9, and 10) and four positively stated items (items 4, 5, 7, and 8) that are measured on a five-point Likert scale. The total score ranges from 0 to 40 with a higher score indicating higher levels of perceived stress [20].

### 2.8. Statistical Analysis

**Figure 2:**
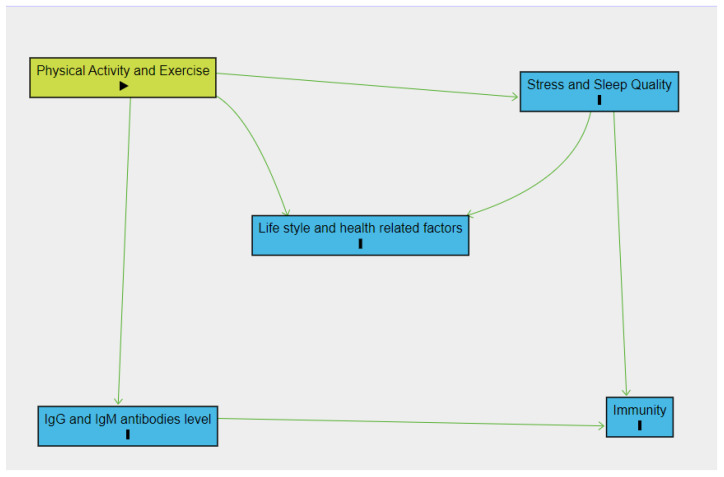
Directed acyclic graph (DAG) demonstrating relationship between physical activity and immunity as well as associated variables.

Descriptive statistics were utilized. Data are expressed as the mean ± standard error of the mean and analyzed by independent samples *t*-tests. Normality of the distribution was verified for all continuous variables by the Kolmogorov–Smirnov test. Comparisons within the groups were performed by using paired *t*-test. Comparisons between the groups were performed using multivariate analysis of variance (MANOVA), adjusting for sleep and stress. *p* < 0.05 was considered statistically significantly different. SPSS version 27 was used for the analysis. Potential confounders (stress and sleep quality) were selected for adjustment based on directed acyclic graphs (DAG) taking into account prior knowledge regarding their associations with physical activity and immunity. Regarding DAG results, stress and sleep quality had to be adjusted for in analyses of the association of physical activity with immunity (Figure 2) [21].

## 3. Results

Forty Health Science Center (HSC) students (eight males and 32 females) participated in this study. Their ages ranged from 18 to 23 years, with a mean age of 20.07 years (SD = 1.654). All of the students received the Pfizer vaccine (Table 2). Table 1 shows the demographic data of the participants: all 40 participants received the Pfizer vaccine; eight (representing 20% of the respondents) were male, while the other 32 (80%) were female; all 40 of the respondents were aged between 18 and 23 years (mean = 20.07); 21 (52.5%) of the respondents were from the Allied Health Faculty, nine (22.5%) were from the Medicine Faculty, eight (20%) were from the Pharmacy Faculty, and two (5%) were from the Dentistry Faculty.

Regarding the differences among the main study variables, it was noted that there were not any significant differences between the control and intervention groups (Table 3).

Likewise, Table 4 shows the statistical paired *t*-test results of the pre and post values of IgG, IgM, sleep quality and stress level outcomes of the control and intervention groups. The table exhibits the mean, the standard deviation (SD), the mean change, the standard error of the mean (SEM), the t value, and the *p* value of the outcome measure. Table 5 highlights comparisons between two groups, control (*n* = 20), and treatment (*n* = 20). The mean value for IgG and IgM antibodies level was higher in the control group, specifically in the IgG antibodies level (*p* < 0.002). The IgM antibodies level also showed the same trend of differences, but with a marginal value (*p* = 0.05). The IgG and IgM antibodies level mean score was not affected by sleep and stress as confounding factors. This was explored after controlling for potential confounding effects of sleep and stress as it is shown in Table 5.

### 3.1. Effects of the Physical Activity of Students Vaccinated with the Third Dose on IgG and IgM Antibody Levels

**Figure 3:**
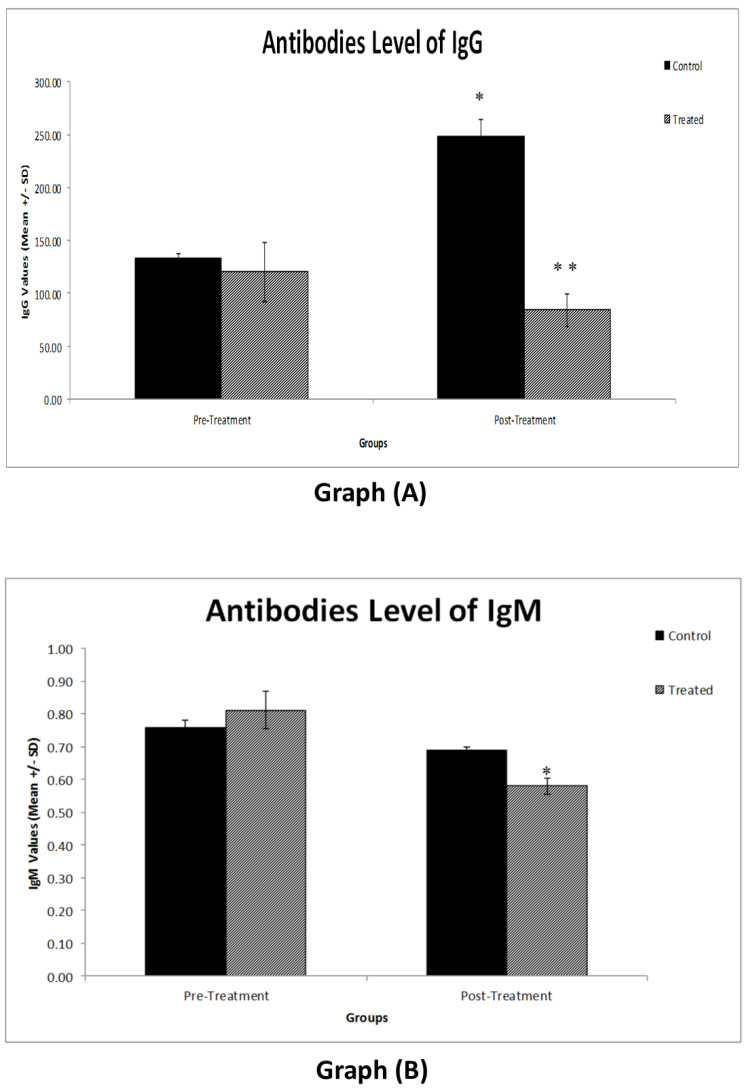
(**A**) Graph comparing the IgG antibody level of the different experimental groups. The control group showed an increased IgG antibody level (* *p* < 0.002), whereas the treatment group showed a continuous decrease in IgG antibody level (** *p* < 0.002). (**B**) Graph comparing the IgM antibody levels of the different experimental groups. The treatment group showed a decrease in IgM antibodies (* *p* < 0.05). The values are the mean ± SE (*n* = 20). Group 1: Control group; Group 2: Treatment group.

Data from Figure 3A demonstrate that there was no significant difference between the two groups before starting the intervention. However, there was a significant (*p* < 0.002) increase in the IgG antibody level in the control group. The IgG antibody level of the treatment group showed a significant decrease compared to the control group (*p* < 0.001). Likewise, the IgM antibody level of all groups did not show any significant difference before starting the intervention. However, there was a significant (*p* < 0.049) decrease in the IgM level of the treatment group after treatment compared to before treatment (Figure 3B).

### 3.2. Effects of the Physical Activity of Students Vaccinated with the Third Dose on Sleep Quality and Stress

**Figure 4:**
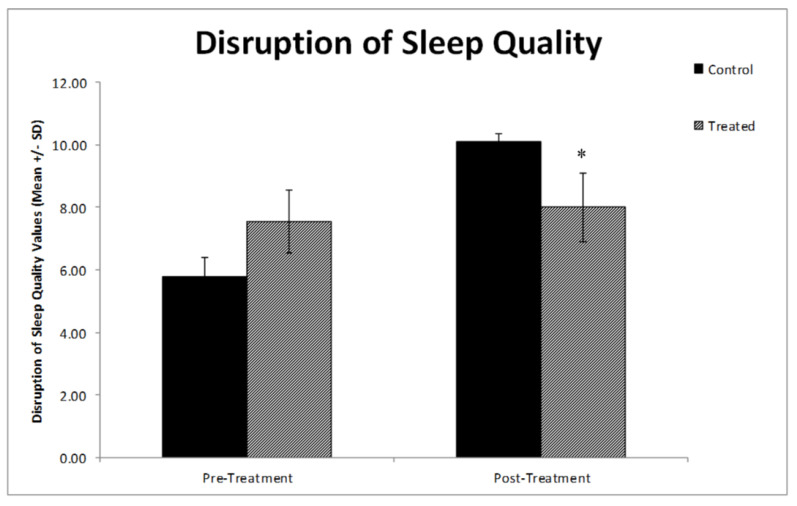
Graph comparing the disruption in sleep quality level of the different experimental groups. The treatment group showed that there was a significant decrease in the disruption of sleep quality level compared to the control group (* *p* < 0.035). The values are the mean ± SE (*n* = 20). Group 1: Control group; Group 2: Treatment group.

**Figure 5:**
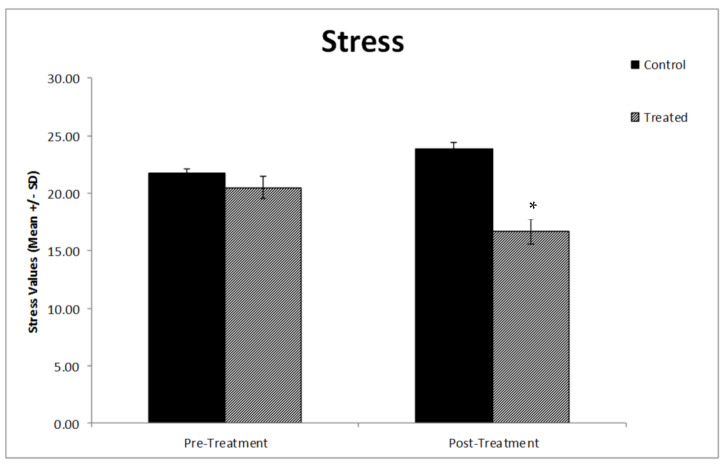
Graph comparing the stress level of the different experimental groups. The stress significantly decreased in the treatment group compared to the control group after the intervention (* *p* < 0.023). The values are the mean ± SE (*n* = 20). Group 1: Control group; Group 2: Treatment group.

Regarding sleep quality, no significant differences between the two groups at baseline were present; however, post-intervention, the analyses showed sleep disruption to be significantly lower in the physically active group compared to the control group (*p* < 0.035) (Figure 4). Likewise, the stress factor also significantly decreased in the exercise group compared to the control group after the intervention (*p* < 0.023) (Figure 5).

## 4. Discussion

### 4.1. Antibody Level

Both the IgG and IgM antibody levels were significantly decreased in the physically active group, while the less active students (control group) had increased levels of IgM and IgG. This may be related to the working mechanism of the Pfizer vaccine, which interacts with the musculoskeletal system, possibly leading to a breakdown of the vaccine itself after elevated levels of physical activity. This finding is in line with a recently published study that reported the working mechanism of the Pfizer vaccine to possibly be compromised by more physically active individuals [22]. That is, an increased level of physical activity could contribute to a reduction in the IgG and IgM antibody levels of the Pfizer COVID-19 vaccine. In addition, our outcome that the physical activity group decreased their antibodies compared to the control group after 4 weeks of intervention might have to do with the acute effects of physical activity on the development of antibodies. For instance, a recent systematic review [23] showed that the immune system is affected by acute exercise in healthy participants, with the specialized regulatory cells that are enclosed in the CD4+ T cells either not altered or decreased following an exercise session. Therefore, it is plausible that acute exercise/physical activity may, at least in the short-term, compromise immune function, although some experts disagree with this point of view [24]. Moreover, Erika and her colleagues indicated that acute exercise decreases vaccine reactions following influenza vaccination among older adults [25]. This could also support our experimental findings pertaining to young Health Science Center (HSC) students. However, another study reported that physically active individuals experience a better immune response compared to inactive individuals upon COVID-19 vaccination [16]. These conflicting findings warrant further high-quality research to investigate the effects of physical activity on vaccines. Future studies with a larger sample size and various vaccination types are needed to better understand this mechanism.

### 4.2. Sleep Quality and Stress

Our study results demonstrated that the physically active group, who had low IgG levels, showed reduced levels of stress and improved quality of sleep. Studies have shown that good sleep quality boosts immunity, while sleep deprivation produces a severe negative effect on immunity [26]. This is further supported by the role of sleep on the frequency of Ag-specific Th cells and IgG level post vaccination in adapting a better immune response and immunological memory [27]. A strong association between sleep quality and immunity within vaccine response has also been reported, where the IgG level was found to determine a longer duration of recovery post vaccination [28]. Lammers-van der Holst showed that the people infected with COVID-19 with higher levels of sleep quality had higher levels of IgG [29]. Such reports confirm the association between sleep quality, vaccination, and level of immune response and support our results, which show that the immune system is boosted through having good sleep quality. This may be a potential factor to decrease the IgG level of the exercise group. This is in agreement with other studies that have reported physical activity to improve sleep quality and accounts for fewer disruptions during sleep [30]. Physical activity has also been found to reduce the students’ stress levels, which is in line with our study findings.

#### 4.2.1. Limitations and Future Directions

The limitations of this study include the small size and the limited behavioral, clinical, and biochemical parameter measurements. It would have also been informative to compare the effects of physical activity with multiple vaccination types. Future studies with various participant age groups and different vaccination types are needed to reach conclusive findings regarding the benefits of COVID-19 vaccination.

#### 4.2.2. Conclusions and Implications

Physically active individuals are expected to have a better immune response and a higher level of antibodies after vaccination, as vaccinations are intended to increase the number of antibodies, while physical activity contributes to better immunity, thereby decreasing stress and improving sleep quality. A COVID-19 booster dose has been administered around the globe to increase immunity and antibody levels in the population. However, such expected findings regarding antibodies after vaccination were not found in the physically active individuals in this study. Further research is necessary to determine the causes behind the significant decrease in IgM and IgG antibodies in physically active participants, and to further understand the magnitude of the decrease in antibodies against the COVID-19 virus in physically active individuals. This study could result in consequences or recommendations applicable to the general population. One the other hand, in future studies, a blood sample may be collected during the physical activity, for example every week. This collection could help to better understand the immune system trend during the sessions. Moreover, salivary samples can also be collected. This might not include an invasive technique in order to collect and evaluate the levels of both immunoglobulins and hormones such as cortisol and testosterone.

## Figures and Tables

**Table 1:** The Borg Rating of Perceived Exertion Scale.

Rating	Perceived Exertion
6	No Exertion
7	Extremely light
8	
9	Very light
10	
11	Light
12	
13	Somewhat hard
14	
15	Hard
16	
17	Very hard
18	
19	Extremely Hard
20	Maximum exertion

**Table 2:** Demographic data of the participants: 40 participants received the Pfizer vaccine; eight (20%) were male, while the other 32 (80%) were female; all 40 were aged between 18 and 23 years 40 (mean = 20.07); all 40 average BMI were 23.1; 21 (52.5%) were from the Allied Health Faculty, nine (22.5%) from the Medicine Faculty, eight (20%) from the Pharmacy Faculty, and two (5%) from the Dentistry Faculty.

Demographics	*N* = 40	Mean SD
**Type of vaccine** Pfizer		
**Gender**		
Male Female	8 (20)% 32 (80)%	
**Age**		
(18–23)	40 (100)%	Mean: 20.07 SD: 1.654
**BMI (Kg/m^2^)**	Mean: 23.1 SD: 3.8	
**Faculities**		
Medicine Allied Health Science Pharmcy Dentistry	9 (22.5)% 21 (52.5)% 8 (20)% 2 (5)%	

**Table 3:** Baseline Characteristics of Participants.

Characteristic	Control (*n* = 20)	Treatment (*n* = 20)	*p*-Value
Mean (±SD) Age	20.07 ± 1.654	21.02 ± 1.421	0.818
% Male	15%	25%	0.442
Mean (±SD) BMI (Kg/m^2^)	23.1 ± 3.8	22.9 ± 3.8	0.61
Mean (Min/day) * Physical activity level	37.1	36.25	0.58
Mean (±SD) Antibodies level of IgG	147.68 ± 4.7	128.5 ± 11.1	0.073
Mean (±SD) Antibodies level of IgM	0.73 ± 0.188	0.81 ± 0.047	0.147
Mean (±SD) Disruption of Sleep Quality	5.87 ± 1.77	7.54 ± 3	0.051
Mean (±SD) Stress level	21.77 ± 0.92	20.5 ± 2.4	0.22

* Physical activity level by using (MET).

**Table 4:** Paired *t*-test comparing pre and post values of IgG, IgM, sleep quality and stress level.

Group	Pair	Outcome Measure	Mean	SD	Mean Change	SEM	t-Value	*p*-Value
Control Group	Pair 1	Pre IgG Post IgG	147.68 248.77	4.7 15.6	101.09	0.04	−3.012 *	0.0001
	Pair 2	Pre IgM Post IgM	0.62 0.69	0.188 0.12	0.07	7.85	−15.89 *	0.007
	Pair 3	Pre sleep quality Post sleep quality	5.87 10.11	1.77 0.99	4.24	0.46	−5.63 *	0.0001
	Pair 4	Pre stress Post stress	21.77 23.88	0.92 1.41	2.11	0.24	−20.78 *	0.0001
Treatment Group	Pair 1	Pre IgG Post IgG	128.5 84.08	11.1 15.19	44.42	12.8	3.29 *	0.004
	Pair 2	Pre IgM Post IgM	0.81 0.61	0.047 0.24	0.2	0.01	4.25 *	0.0001
	Pair 3	Pre sleep quality Post sleep quality	7.54 8.1	3 1.44	0.56	0.42	−3.955 *	0.001
	Pair 4	Pre stress Post stress	20.5 16.66	2.4 1.99	3.87	0.85	0.989 *	0.025

* *p*-value < 0.05. SD, standard deviation: SEM, standard error of the mean.

**Table 5:** Multivariate analysis between groups adjusted for sleep and stress.

Variables	Total (*n* = 40)	Control (*n* = 20)	Treatment (*n* = 20)	Group Effect *p*-Value	Sleep-Adjusted *p*-Value	Stress Adjusted *p*-Value	Sleep and Stress Adjusted *p*-Value
**IgG antibodies level**	8.98 (18.3)	248.77 (15.6)	84.08 (15.9)	0.002 *	0.540	0.782	0.850
**IgM antibodies level**	0.04 (0.035)	0.69 (0.12)	0.61 (0.24)	0.05 *	0.164	0.174	0.088

* *p* < 0.05. significant differences between-subject effects.

## Data Availability

Not applicable.

## References

[1] Rakhsha A., Azghandi S., Taghizadeh-Hesary F. (2020). COVID-19 pandemic and patients with cancer: The protocol of a Clinical Oncology center in Tehran, Iran. Rep. Pract. Oncol. Radiother..

[2] Akbari H., Taghizadeh-Hesary F., Bahadori M. (2022). Mitochondria determine response to anti-programmed cell death protein-1 (anti-PD-1) immunotherapy: An evidence-based hypothesis. Mitochondrion.

[3] Woods J.A., Hutchinson N.T., Powers S.K., Roberts W.O., Gomez-Cabrera M.C., Radak Z., Berkes I., Boros A., Boldogh I., Leeuwenburgh C. (2020). The COVID-19 pandemic and physical activity. Sports Med. Health Sci..

[4] Ranasinghe C., Ozemek C., Arena R. (2020). Exercise and well-being during COVID 19—Time to boost your immunity. Expert Rev. Anti-Infect. Ther..

[5] Gupta R., Grover S., Basu A., Krishnan V., Tripathi A., Subramanyam A., Nischal A., Hussain A., Mehra A., Ambekar A. (2020). Changes in sleep pattern and sleep quality during COVID-19 lockdown. Indian J Psychiatry.

[6] Crowe J.E., Remington J.S., Klein J.O., Wilson C.B., Nizet V., Maldonado Y.A. (2011). CHAPTER 38—Prevention of Fetal and Early Life Infections Through Maternal–Neonatal Immunization. Infectious Diseases of the Fetus and Newborn.

[7] Wong G.C.L., Narang V., Lu Y., Camous X., Nyunt M.S.Z., Carre C., Tan C., Xian C.H., Chong J., Chua M. (2019). Hallmarks of improved immunological responses in the vaccination of more physically active elderly females. Exerc. Immunol. Rev..

[8] Engler R.J.M., Nelson M.R., Klote M.M., VanRaden M.J., Huang C.-Y., Cox N.J., Klimov A., Keitel W.A., Nichol K.L., Carr W.W. (2008). Half- vs. Full-Dose Trivalent Inactivated Influenza Vaccine (2004–2005): Age, Dose, and Sex Effects on Immune Responses. Arch. Intern. Med..

[9] Kohut M.L., Cooper M.M., Nickolaus M.S., Russell D.R., Cunnick J.E. (2002). Exercise and Psychosocial Factors Modulate Immunity to Influenza Vaccine in Elderly Individuals. J. Gerontol. Ser. A.

[10] Lange T., Perras B., Fehm H.L., Born J. (2003). Sleep Enhances the Human Antibody Response to Hepatitis A Vaccination. Psychosom. Med..

[11] Hawker G.A., French M., Waugh E., Gignac M., Cheung C., Murray B. (2010). The multidimensionality of sleep quality and its relationship to fatigue in older adults with painful osteoarthritis. Osteoarthr. Cartil. OARS Osteoarthr. Res. Soc..

[12] Maynard S., Kao R., Craig D. (2015). Impact of personal protective equipment on clinical output and perceived exertion. J. R. Army Med. Corps.

[13] Bai Y., Tompkins C., Gell N., Dione D., Zhang T., Byun W. (2021). Comprehensive comparison of Apple Watch and Fitbit monitors in a free-living setting. PLoS ONE.

[14] Hallam J., Jones T., Alley J., Kohut M.L. (2022). Exercise after influenza or COVID-19 vaccination increases serum antibody without an increase in side effects. Brain Behav. Immun..

[15] Edwards K.M., Pung M.A., Tomfohr L.M., Ziegler M.G., Campbell J.P., Drayson M.T., Mills P.J. (2012). Acute exercise enhancement of pneumococcal vaccination response: A randomised controlled trial of weaker and stronger immune response. Vaccine.

[16] Bortolini M., Petriz B., Mineo J., Resende R. (2022). Why Physical Activity Should Be Considered in Clinical Trials for COVID-19 Vaccines: A Focus on Risk Groups. Int. J. Environ. Res. Public Health.

[17] Buysse D.J., Reynolds C.F., Monk T.H., Berman S.R., Kupfer D.J. (1989). The Pittsburgh Sleep Quality Index: A new instrument for psychiatric practice and research. Psychiatry Res..

[18] Kelly K.S., Taylor D.J., Kohut M.L., Hallam J., Song K. (2014). 8. Stress, insomnia and influenza vaccine response. Brain Behav. Immun..

[19] Liu X., Zhao Y., Li J., Dai J., Wang X., Wang S. (2020). Factor Structure of the 10-Item Perceived Stress Scale and Measurement Invariance Across Genders Among Chinese Adolescents. Front. Psychol..

[20] Tabassum M., Parvej M., Ahmed F., Zafreen F., Sultana S. (2021). Effect of COVID-19 on Perceived Stress among Bangladeshi People. Ment. Health Rev. J..

[21] Textor J., van der Zander B., Gilthorpe M.S., Liskiewicz M., Ellison G.T. (2016). Robust causal inference using directed acyclic graphs: The R package ‘dagitty’. Int. J. Epidemiol..

[22] Mascellino M.T., Di Timoteo F., De Angelis M., Oliva A. (2021). Overview of the Main Anti-SARS-CoV-2 Vaccines: Mechanism of Action, Efficacy and Safety. Infect. Drug Resist..

[23] Proschinger S., Winker M., Joisten N., Bloch W., Palmowski J., Zimmer P. (2021). The effect of exercise on regulatory T cells: A systematic review of human and animal studies with future perspectives and methodological recommendations. Exerc. Immunol. Rev..

[24] Campbell J.P., Turner J.E. (2018). Debunking the Myth of Exercise-Induced Immune Suppression: Redefining the Impact of Exercise on Immunological Health Across the Lifespan. Front. Immunol..

[25] Bohn-Goldbaum E., Pascoe A., fiatarone singh M., Singh N., Kok J., Dwyer D., Mathieson E., Booy R., Edwards K. (2020). Acute exercise decreases vaccine reactions following influenza vaccination among older adults. Brain Behav. Immun.-Health.

[26] Bryant P.A., Trinder J., Curtis N. (2004). Sick and tired: Does sleep have a vital role in the immune system?. Nat. Rev. Immunol..

[27] Lange T., Dimitrov S., Bollinger T., Diekelmann S., Born J. (2011). Sleep after Vaccination Boosts Immunological Memory. J. Immunol..

[28] Spiegel K., Sheridan J., Van Cauter E. (2002). Effect of Sleep Deprivation on Response to Immunizaton. JAMA J. Am. Med. Assoc..

[29] Lammers-van der Holst H.M., Lammers G.J., van der Horst G.T.J., Chaves I., de Vries R.D., GeurtsvanKessel C.H., Koch B., van der Kuy H.M. (2022). Understanding the association between sleep, shift work and COVID-19 vaccine immune response efficacy: Protocol of the S-CORE study. J. Sleep Res..

[30] Wang F., Boros S. (2019). European Journal of Physiotherapy The effect of physical activity on sleep quality: A systematic review Feifei Wang & Szilvia Boros The effect of physical activity on sleep quality: A systematic review. Eur. J. Physiother..

